# Non-Linear Elasticity of Extracellular Matrices Enables Contractile Cells to Communicate Local Position and Orientation

**DOI:** 10.1371/journal.pone.0006382

**Published:** 2009-07-24

**Authors:** Jessamine P. Winer, Shaina Oake, Paul A. Janmey

**Affiliations:** 1 Department of Bioengineering, University of Pennsylvania, Philadelphia, Pennsylvania, United States of America; 2 Institute for Medicine & Engineering, University of Pennsylvania, Philadelphia, Pennsylvania, United States of America; 3 Department of Physiology, University of Pennsylvania, Philadelphia, Pennsylvania, United States of America; Dalhousie University, Canada

## Abstract

Most tissue cells grown in sparse cultures on linearly elastic substrates typically display a small, round phenotype on soft substrates and become increasingly spread as the modulus of the substrate increases until their spread area reaches a maximum value. As cell density increases, individual cells retain the same stiffness-dependent differences unless they are very close or in molecular contact. On nonlinear strain-stiffening fibrin gels, the same cell types become maximally spread even when the low strain elastic modulus would predict a round morphology, and cells are influenced by the presence of neighbors hundreds of microns away. Time lapse microscopy reveals that fibroblasts and human mesenchymal stem cells on fibrin deform the substrate by several microns up to five cell lengths away from their plasma membrane through a force limited mechanism. Atomic force microscopy and rheology confirm that these strains locally and globally stiffen the gel, depending on cell density, and this effect leads to long distance cell-cell communication and alignment. Thus cells are acutely responsive to the nonlinear elasticity of their substrates and can manipulate this rheological property to induce patterning.

## Introduction

Over the last decade it has been demonstrated that a variety of tissue-forming cells can both sense the stiffness of their substrate and apply a controlled force onto that substrate. Not all cell types respond to stiffness changes in the same way, but many including endothelial cells [1], fibroblasts [2], mammary epithelial cells [3] astrocytes [4], macrophages [5] and mesenchymal stem cells [6], exhibit increased spreading and adhesion on stiffer substrates compared to softer ones. Numerous proteins and protein complexes required for responding to substrate stiffness, such as the actomyosin network, small GTPases, protein phosphatases, and integrin adhesion sites have been identified, but the mechanism by which forces govern the interactions among these proteins are not yet defined.

Mechanically compliant materials for studying cellular responses to substrate stiffness are typically made from synthetic polymers whose elastic moduli are independent of applied strain and are determined by polymer and crosslinker density. The polyacrylamide gel system developed by Pelham and Wang allows the substrate's physical properties to be manipulated without affecting its chemical properties [7]. Adhesion molecules are covalently attached to the gel's surface after polymerization, resulting in a uniform coverage regardless of gel stiffness. Similar gels have been adapted for traction microscopy to quantify the forces that cells exert on compliant substrates [8], and the linearity of the elastic response is essential to the algorithms that permit forces to be calculated from the measured displacement fields. Other synthetic systems with linear elasticity include silicone films [9] and flexible PDMS micropillars [10].

Studies done on synthetic gels have been used to understand how cells respond to the mechanical properties of the tissue microenvironment; however, extracellular matrix proteins such as collagen type I and fibrin display nonlinear mechanical properties such as strain stiffening [11] and negative normal stress [12]. In these materials the elastic modulus of the gel increases orders of magnitude as the applied strain increases such that the resistance that a cell feels would be a strong function of the strain that it applies. Many cell types also modulate the force they apply according to the stiffness of the gel, applying smaller forces when cultured on softer gels [13]. Using responses to linearly elastic materials to predict a cell's behavior on nonlinear gels is further complicated since it is not known what property the cell's mechanosensor is measuring. For example, whether cells attempt to exert a constant deformation and monitor the required stress, or whether they exert a constant stress and respond to the degree of strain remains an open question [14].

Strain stiffening is a property rarely seen in synthetic polymers but is common among gels made from filamentous biological polymers such as fibrin, collagen and actin. As it is a property prevalent in extracellular matrix proteins, it has been postulated that strain stiffening evolved to protect tissues from tearing under large stresses but it could also play a role in tissue development, homeostasis and repair. The results presented here show that contractile, durotactic cells such as NIH 3T3 fibroblasts and hMSCs are acutely responsive to the nonlinear properties of their substrate and respond to the material's high strain modulus. On fibrin with a low strain modulus of 100 Pa the cells spread as if on a much stiffer gel using actomyosin contraction to strain the fibrin gel, locally increase its modulus, and achieve optimal spreading through a force-limited mechanism. Local strain stiffening allows an initially isotropic matrix to reinforce cell-applied mechanical anisotropy and transmit forces between cells up to half a millimeter apart. In this way isolated cells can create far-reaching mechanical gradients and produce a global pattern, a phenomenon potentially related to pattern formation during wound healing or tissue development.

## Results

### Cells cultured on soft fibrin spread as though on a stiff substrate

**Figure 1:**
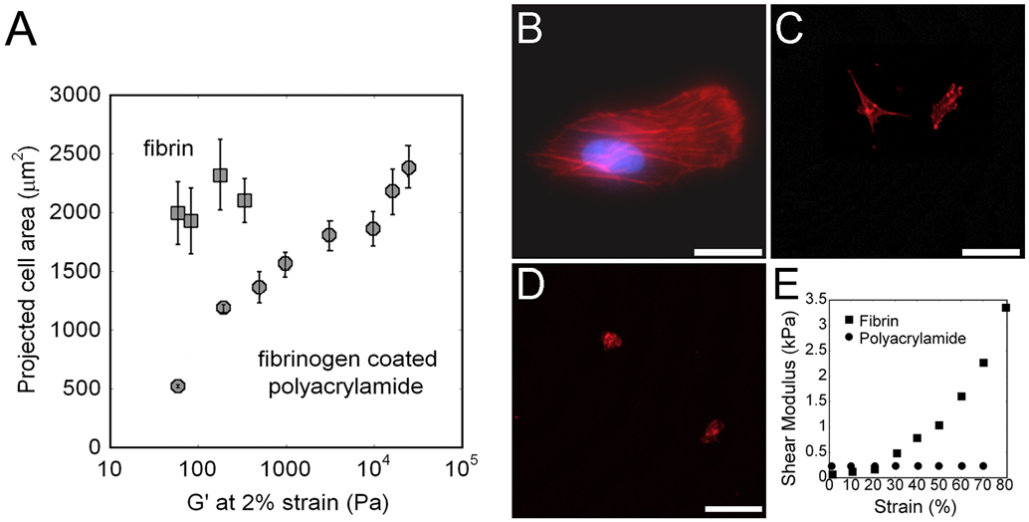
Spread area of cells on fibrin is independent of the gel's low strain modulus. A) Fibroblast's spread area on fibrin (▪) or fibrinogen coated polyacrylamide (•) gels of varying stiffnesses after 18 hours on substrate. Reported as mean±SE, n = 3, at least 50 cells per group. Fluorescence image of actin (red) structures of fibroblasts on 2 mg/ml fibrin Scale bar = 20 µm, Nucleus in blue.(B), 10 kPa fibrinogen coated polyacrylamide Scale bar = 100 µm (C) and 0.1 kPa fibrinogen coated polyacrylamide Scale bar = 100 µm (D). E) Strain versus shear modulus, measured by oscillating rheology, of 2 mg/ml fibrin (▪) or 7% acrylamide, 0.05% bisacrylamide gels (•).

Consistent with previous reports [1] fibroblasts are round on fibrinogen-coated polyacrylamide gels with a shear modulus of 100 Pa and become increasingly spread as the gel stiffness increases until they reach a spread area of approximately 2200 µm^2^ on 16 kPa polyacrylamide gels (Fig. 1A). In contrast, on 1 mm thick fibrin gels of 1, 2, 4 and 8 mg/ml, with low strain shear moduli of 30, 60, 140 and 350 Pa respectively, the fibroblasts spread to areas statistically similar to each other and to the average spread area on the stiffest fibrinogen-coated polyacrylamide gel. Similar results were obtained using human mesenchymal stem cells (hMSCs) on the two kinds of substrates. Cells on fibrin displayed actin stress fibers, which are noticeably absent in cells cultured on soft polyacrylamide (Fig. 1B, C and D).

One possible explanation for this response is that the cells deform the fibrin gels enough to sense the high strain modulus rather than the low strain modulus of the substrates. The shear modulus of polyacrylamide is independent of strain whereas the modulus of fibrin increases as the applied strain increases (Fig. 1E) [11]. Therefore, if application of a small strain to the substrate is sufficient for the cells to respond to substrate mechanics, then 2 mg/ml fibrin and 70 Pa polyacrylamide gels will appear equally soft, but if the cells apply strains large enough to enter the strain-stiffening regime of fibrin elasticity, then they will respond to fibrin as a stiffer matrix than polyacrylamide. The latter hypothesis is consistent with the data, for figure 1E shows that the modulus of 2 mg/ml fibrin at 80% strain is 3.7 kPa and the average spread area of cells on these gels is ∼1900 µm^2^; this is comparable to cells on a 3.2 kPa fibrinogen-coated polyacrylamide gel which have an average spread area of ∼1800 µm^2^.

### A network of fibers is required for cells to spread on fibrin

**Figure 2:**
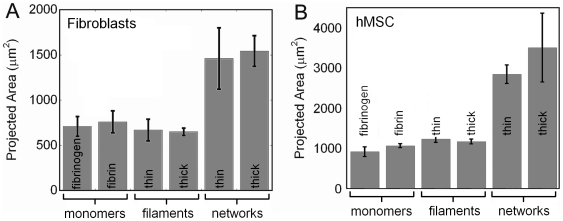
Cells spread on “soft” polyacrylamide only if coated with a continuous fibrin network. Spread area of fibroblasts (A) or hMSCs (B) cultured for 18 hours on 100 Pa polyacrylamide gels coated with either fibrinogen monomers, fibrin monomers, thin fibers, thick fibers, a network of thin fibers or a network of thick fibers. Reported as mean±SE, n = 3, at least 50 cells per group.

In addition to the network mechanical properties there are several other differences between fibrinogen-coated polyacrylamide and fibrin gels: ligand conformation, ligand density, ligand orientation and individual polymer mechanical properties. To test if these factors account for the different morphologies on fibrin gels and fibrinogen-coated polyacrylamide, the average spread area of cells was measured after incubation on 100 Pa polyacrylamide gels coated with fibrinogen monomers, fibrin monomers, a non contiguous coating of thin fibrin fibers, a non contiguous coating of thick fibrin fibers, a continuous network of thin fibers, or a continuous network of thick fibers. Only the gels coated with a fibrin network induced cell spreading, and the same trend occurred regardless of cell type (Fig. 2A and 2B). Since the fibrin monomers and filaments all have higher tensile moduli than the gel but are linearly elastic [15], [16], [17] this result bolsters the argument that the fibrin gel's nonlinear mechanical properties, rather than their shape or chemical properties, induce cell spreading.

Another factor that could affect cell morphology is ligand density, which could not be independently controlled under the experimental conditions that alter fibrin stiffness; however, analysis of scanning electron micrographs (Fig. S1) of the substrate surface does not support this hypothesis. These images were used to estimate the fractional surface coverage of the gel and indicated that monomer-coated substrates were 30% covered, filament-coated substrates were 50% covered and network-coated substrates were 100% covered. If ligand density were the controlling factor, then one would expect that the filament substrates would have displayed an intermediate projected cell area. They have both an increased local linear density from the presence of fibers and an increased global density. Since the spread area on the filament-coated substrates is not significantly different from that on the monomer-coated substrates, it is unlikely that ligand density or activation of a wound healing response is responsible for the dramatic increase of spread area upon the gels coated with a network.

### Cell applied displacements are cell type, distance, and fibrin gel stiffness dependent

**Figure 3:**
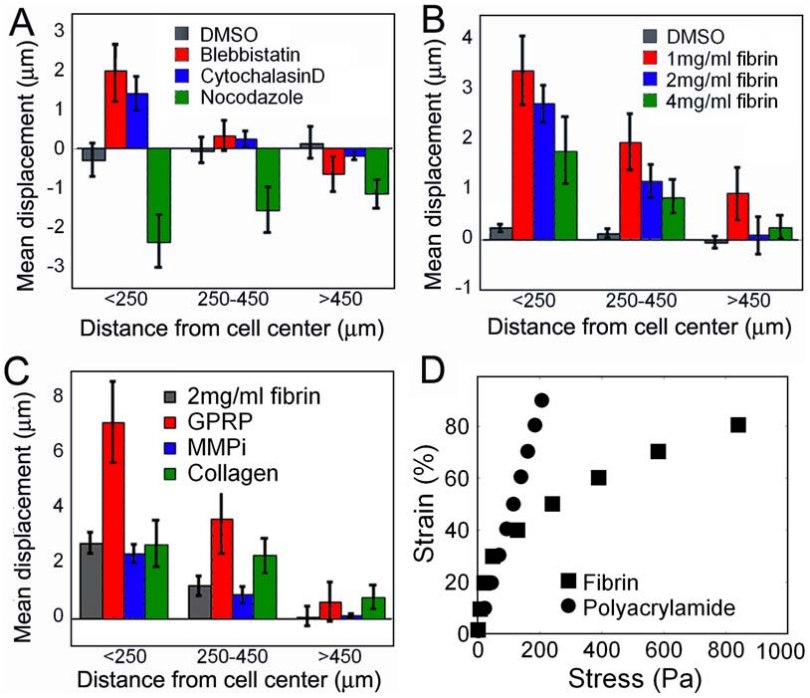
Cell applied tractions are cell type, distance and fibrin gel stiffness dependent. Average bead displacement applied by fibroblasts (A) or hMSCs (B, C) as a function of distance from the cell's center. Cytochalasin D was used to relax hMSCs in B. and C. Reported as mean±SE, n = 5 cells per condition, at least 80 beads counted in each zone. D) Stress versus strain curve, measured by oscillating rheology, of 2 mg/ml fibrin (▪) or 7% acrylamide, 0.05% bisacrylamide gels (•).

**Figure 4:**
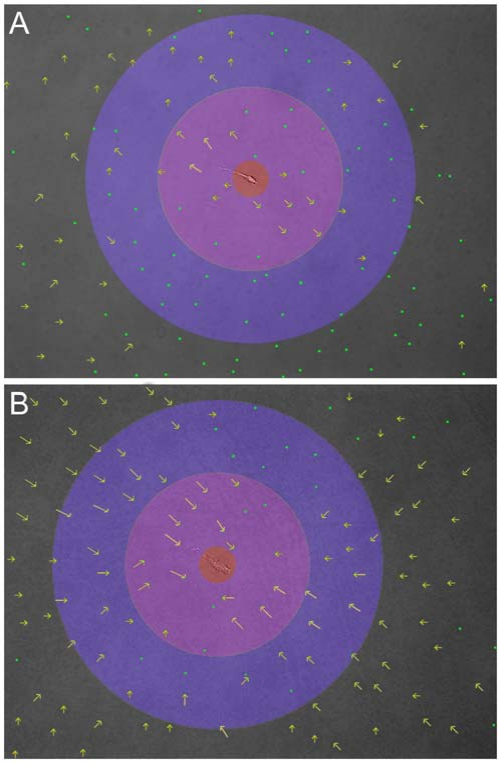
Cells apply asymmetric strain fields. Example displacement patterns of fibroblasts on 2 mg/ml fibrin gels treated with either blebbistatin (A) or nocodazole (B). To allow for visualization in this format only 1/10^th^ of all tracked beads are shown and displacements have been magnified 5× and indicated by the arrows which point in direction of bead movement after drug treatment. The red circle has a radius of 50 µm. The radius of the purple circle is 250 µm and the radius of the blue circle is 450 µm.

To study the relationship between cell adhesion forces and network structure, cells were cultured on fibrin gels coated with fluorescent beads, and the contraction or relaxation of the gel was monitored by tracking bead displacements in response to drug treatments. On 2 mg/ml fibrin, fibroblasts, which are approximately 50 µm in diameter when spread, displaced beads an average of 1.5 µm up to 250 µm away from the cell's centroid (Fig. 3A and 4A), and the 100 µm diameter hMSCs displaced beads up to 450 µm away (Fig. 3B). This displacement was determined by treating the cells with either blebbistatin, to inactivate force generation by non muscle myosin II, or with cytochalasin D, which disassembles the actin fibers that myosin acts upon. Both treatments resulted in a similar pattern of bead displacements, consistent with an effect on acto-myosin contractility. Treating fibroblasts with nocodazole, a microtubule destabilizing drug, resulted in the cells increasing the applied force and drawing closer most beads in the field of view (Fig. 3A and 4B). This nocodazole-induced contraction is consistent with previous reports that depolymerization of microtubules stimulates myosin light chain phosphorylation and increased contractility [18]. The DMSO control had no significant effect on the position of the beads. The responses to drug treatments reported here are consistent with previous traction force microscopy on synthetic gels [19], [20].

The hMSCs applied stronger active tractions, possibly because their larger spread area allowed them to engage a larger number of integrins, allowing for a more dynamic range. Figure 3B shows that the concentration of fibrin also affected the degree of bead displacement, with displacements increasing as fibrin concentration decreases. Since both stiffness and ligand density increase with increasing fibrinogen concentration, the polymerization inhibitor GPRP was added to a 2 mg/ml gel to lower the low strain modulus to 30 Pa, below that of a 1 mg/ml gel, without changing the ligand density [21]. Under these conditions the hMSCs produced even larger average displacements, confirming that stiffness and not ligand density plays the dominant role in determining the size of the displacements (Fig. 3C). This result fits with the hypothesis that the cells exploit the strain-stiffening properties of the material to achieve a specific substrate resistance, since the lower the initial modulus, the more the cell must displace the beads in order to achieve the same final stiffness.

Since fibrin is a 3D matrix, which cells can penetrate and invade during the overnight incubation, it is possible that the observed behavior is a result of encountering a three dimensional environment. Since matrix metalloprotease activity is required for cell invasion of fibrin *in vitro* [22], [23], cells were cultured on a 2 mg/ml fibrin gel with the broad spectrum matrix metalloprotease inhibitor (MMPi) GM6001 added to the media. The cell protrusions were narrower, but was no significant effect on the measured displacements (Fig. 3C).

To examine the effect of other ECM proteins that engage different integrins on the cell's applied forces, gels were incubated with monomeric type 1 collagen prior to the addition of cells. Figure 3C shows that adsorbing collagen monomers to the fibrin gel has no effect on the displacement of beads close to the cell boundary, but it increases the displacement of beads farther than 250 µm from the cell center. One interpretation of these results is that adding collagen increases the stress threshold that the hMSCs achieve either by chemically stimulating an increase in applied force, or by engaging a larger number of integrins, since fibrin and collagen bind separate populations of integrins. Type I collagen is engaged through the alpha1/beta1 and alpha2/beta1 integrin pairs [24] whereas fibrin gels are reportedly engaged through the alphav/beta3 integrin pair [25], [26]. Due to fibrin's strain stiffening, stress and strain are not linearly related, and in regions where the gel is already highly stressed increases in applied stress result in negligible strain increases but in regions where the gel was only slightly prestressed the strain increases can be significant (Fig. 3D).

Although the bead displacement is reported as an average over all points within a certain distance from the cell, the displacement field is not uniform around the cell. The displacement field is typically the same shape as the cell, with the largest displacements closest to the cell edge that is farthest from the cell's center (Fig. 4). When myosin is inactivated by blebbistatin, beads as far away as 400 µm are seen to relax away from the cell, with the majority of moving beads oriented along the long axis of the cell (Fig. 4A). These experiments only reveal the recoverable, elastic component of the cell-applied strains and not any permanent rearrangements due to mechanical creep or chemical modifications of the matrix that occur during the prolonged application of force. After nocodazole treatment which acutely hyperactivates acto-myosin contractility, significant matrix displacements are observed more than 500 µm from the cell in the direction of the cell's long axis (Fig. 4B). The greater magnitude and range of these deformations might result both from the lack of matrix relaxation and the fact that the increased contractility triggered by MT depolymerization is exerted on a fibrin matrix that has already been stiffened and aligned by the cell at steady state.

### Global shear modulus of fibrin increases when contractile cells are embedded

**Figure 5:**
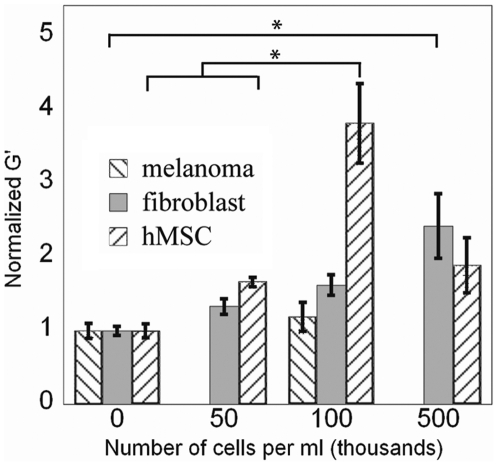
Embedded contractile cells stiffen the shear modulus of a 3D gel. Elastic shear modulus measured by rheology of fibrin gels cultured for 18 hours with varying densities of cells. Reported as mean±SE, n = 3, 8 measurements per sample, *: p<0.001.

To determine if the cell-applied strains are sufficient to globally stiffen a fibrin gel, cells were suspended in polymerizing fibrin and allowed to spread for 18 hours before the low strain shear modulus of gels was measured. Both fibroblasts and hMSCs stiffened the gels in a cell density dependent manner, and at low concentrations hMSC's stiffened the gel to a greater degree than the smaller fibroblasts (Fig. 5). This result is consistent with the finding in figure 3 that hMSC's apply larger forces than fibroblasts. One anomaly in the data is that gels with 500,000 hMSCs/ml had a lower modulus that those with 100,000 hMSCs/ml, possibly because hMSCs are large cells compared to fibroblasts and at such a high density they may disrupt the fibrin network structure. As a control, melanoma cells, which bind but do not spread well in 3D fibrin, did not increase the gel's stiffness when embedded and cultured within fibrin gels. This result shows that the cells must actively contract the matrix to stiffen it.

### Atomic force microscopy confirms that cell-applied displacements locally stiffen fibrin

**Figure 6:**
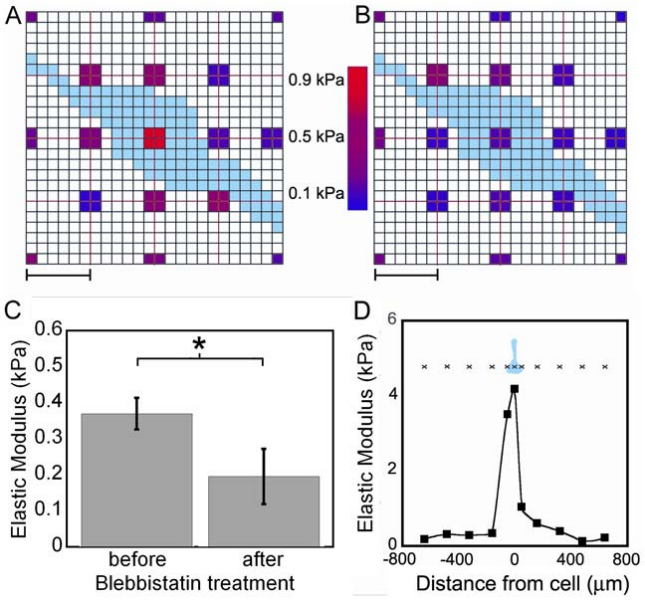
The cell applied strains are creating local stiffness gradients. AFM generated stiffness maps of a fibroblast (in blue) on a 2 mg/ml fibrin gel before (A) and after (B) blebbistatin treatment. Scale bar = 30 µm. The center measurement is of the cell stiffness not the gel stiffness. C) Average tensile modulus of a 2 mg/ml fibrin gel 10–15 µm from the cell's edge before and after blebbistatin treatment. Reported as mean±SD, 3 measurements per cell, 3 cells in total, *: p<0.01 using a paired students t-test D) A stiffness line scan of a hMSC (in blue) on a fibrin gel. Reported stiffness is an estimated tensile modulus. Similar results were seen in independent experiments.

To examine local effects on gel stiffness by cell-applied strains, the atomic force microscope was employed to generate a stiffness map of a 2 mg/ml fibrin gel around a spread fibroblast (Fig. 6A). The gel is clearly stiffer closer to the cell than at the periphery. At the map's periphery the measured Youngs modulus is slightly above 100 Pa and is comparable to the low strain modulus measured by conventional rheometry. After the cell is treated with blebbistatin, both the modulus of the cell and the modulus of the surrounding gel drop significantly (Fig. 6B, 6C). This drop in gel modulus with inactivation of non-muscle myosin confirms that the cellular forces are responsible for locally stiffening the gel. The hMSCs are too large to fit in the scan window of the AFM, so instead points along a line were manually selected. The line scan in figure 6D shows that, as with fibroblasts, hMSCs also locally stiffen the gel, and consistent with figure 3 the effects of the hMSCs persist further away from the cell whereas the stiffness increases by fibroblasts are smaller and taper off more rapidly. Both of these cell types create stiffness gradients that persist beyond their periphery.

### Fibrin substrates induce elongation and patterning in nearby cells

Figures 3 and 6 show that the cell-applied displacements create a strain field that changes the structure and mechanics of the fibrin gel and persists hundreds of microns away from the cell's edge. AFM reveals that prior to cell attachment the gel is an isotropic mesh of thin fibers that vary from 40–90 nm in diameter (Fig. S2A), the high strains applied by the cells appear to bundle and align the fibers perpendicular to the cell's membrane (Fig. S2B) creating a zone that is mechanically and structurally anisotropic. Previous work has shown that substrate mechanosensitive cells display stiffness gradient directed movement or durotaxis [27] toward the stiffer portion of a substrate. To determine what, if any, effect this strain field has on cells caught within it, cell alignment, area, orientation and circularity were analyzed as a function of distance to the membrane of the nearest cell. Membrane to membrane distance was measured because previous experiments have shown that the strongest cell forces, and thus greatest strains, are applied through focal adhesions at the cell's periphery [28]. Cells on polyacrylamide gels of comparable low strain modulus to fibrin are round and thus display no orientation or alignment, making soft linear elastic gels an unsuitable control. As the cells appear to spread in accordance with a stiffer modulus, a 16 kPa fibrinogen-coated polyacrylamide gel was chosen as the control because hMSCs on this substrate had the same average spread area as those on fibrin.

**Figure 7:**
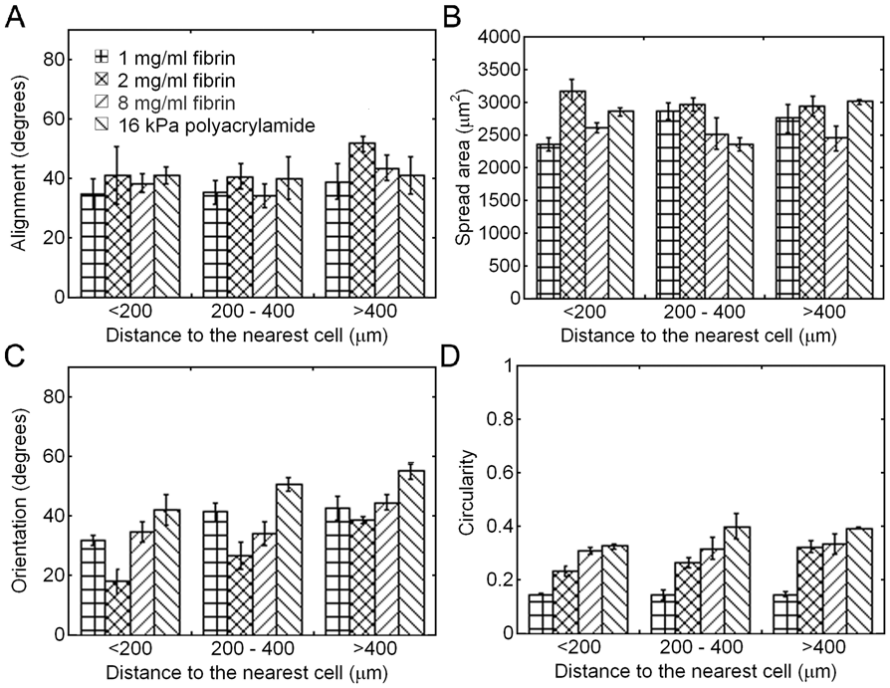
Fibrinogen concentration and distance to the nearest cell both regulate cell shape and patterning. The degree of alignment (A), projected cell area (B), orientation angle (C) and circularity (D) of neighboring hMSCs were analyzed as a function of distance to the nearest cell on four substrates: 1, 2, and 8 mg/ml fibrin as well as 16 kPa fibrinogen coated polyacrylamide. Reported as mean±SE, n = 3, at least 10 cells per group.

Cell alignment, defined as the angle between the long axis of a cell and the long axis of its nearest neighbor, and projected cell area were independent of the distance between cells and the fibrinogen concentration (Fig. 7A and 7B). For cells cultured on 1 and 2 mg/ml fibrin gels, cell orientation angle, defined as the angle between the long axis of the cell and the shortest line to the nearest cell, decreased as the distance between the two cells decreased (Fig. 7C). The range over which this effect occurs corresponds to the range over which hMSCs displaced beads in the earlier experiments. Cells on 8 mg/ml fibrin display a similar trend, but the populations are not statistically different from each other, although they are significantly more oriented to each other than cells on polyacrylamide.

One explanation for why the orientation response was stronger on 2 mg/ml gels than 1 mg/ml gels is that the extremely elongated shape of the cells on 1 mg/ml fibrin, and thus the shape of the resulting strain field reduced the probability that a second cell would come in contact with the affected gel (Fig. 7D, S3). In general, cells on fibrin display an increase in axial ratio and a decrease in circularity, a compactness shape factor defined as 4π (area)/(perimeter)^2^, with a decrease in fibrinogen concentration. The circularity is 1 for a circle and decreases toward 0 as the cell becomes more elongated or stellate in morphology. As a cell engages the surface it will bind to a finite number of integrins and apply an initial force at a few sites. This initial deformation of the fibrin gel will increase the stiffness of that section of the gel over the surrounding gel. Cells have been shown to be sensitive to variations in stiffness and spread towards the stiffer region [27]; therefore, the cell would elongate along these stiffened regions applying stronger tractions at the same time. In this way any initial anisotropy in cell orientation would be reinforced, leading to more elongated cells on the strain stiffening substrate.

**Figure 8:**
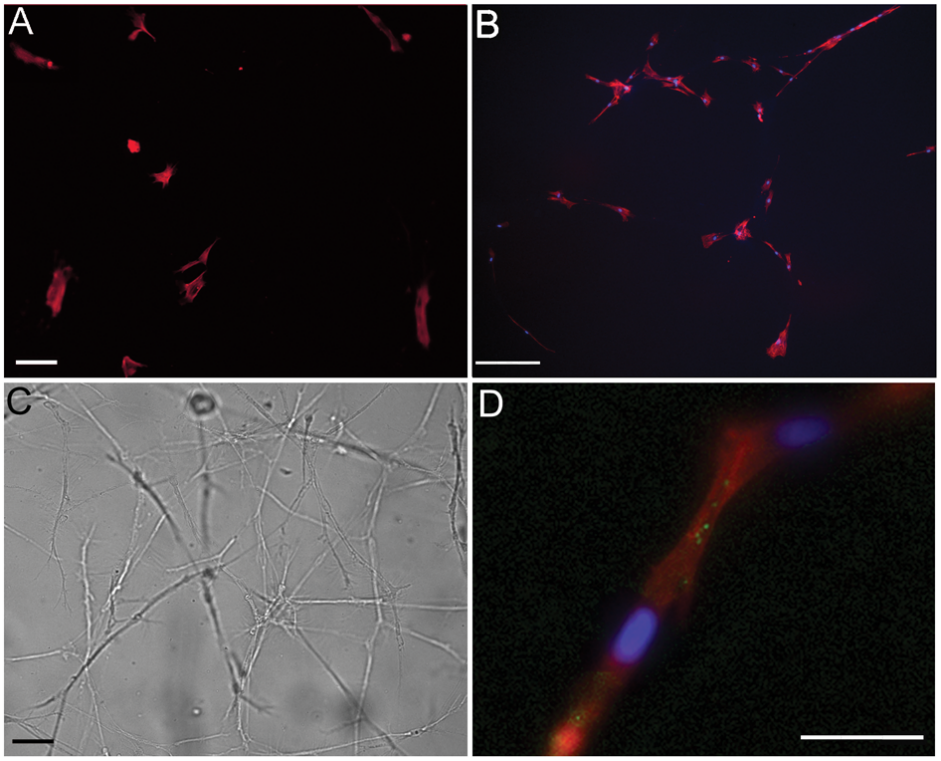
Fibrin gels stimulate cell patterning in 2D and 3D. Characteristic fluorescence image of F-actin structures in hMSCs cultured for 4 days on 16 kPa fibrinogen coated polyacrylamide (A) or 2 mg/ml fibrin (B). C) A bright field image of hMSCs cultured in 3D for 7 days in a 2 mg/ml gel. D) A fluorescently stained junction between two hMSCs in a 3D fibrin gel. For all images F-actin is labeled in red, the nucleus in blue and connexin 43 in green. Scale bar = 100 µm for all images.

Together these elongation and orientation responses contribute to patterning of multicellular ring complexes on fibrin gels in 2D (Fig. 8B) and 3D (Fig. 8C) not seen on uniform linear elastic surfaces (Fig. 8A). By propagating the mechanical signal, the strain stiffening network allows initially isolated cells to make contact and form a chemically connected network through gap junctions (Fig. 8D). These ring structures are reminiscent of images of substrate compliance-dependent capillary morphogenesis of endothelial cells seen using other strain stiffening materials [29], [30], [31]. In this way, the properties of durotactic cells and strain stiffening gels may combine to facilitate patterning of tissues during wound healing.

## Discussion

The mechanical interactions between cells and their substrates have primarily been investigated using synthetic, linearly elastic materials. When studies have been done using biological materials the results have been interpreted assuming that these materials behave similarly to their synthetic counterparts [32]. Some cell types, such as neurons, do appear to respond similarly to the low strain elastic modulus of PA and fibrin gels [4]. This similar response is likely to occur because neurons apply very small forces to their substrate [33], [34] and therefore sense only the low-strain linear elastic modulus. The work presented here demonstrates that mesenchymal cells are acutely responsive to the nonlinear properties of their substrates. Both NIH 3T3 fibroblasts and human mesenchymal stem cells strongly contract fibrin gels to locally stiffen them enough so that they can achieve optimal spreading through a force-limited mechanism. This spreading response is not due to changes in the chemical nature of the substrate or the stiffness of the individual fibrils. By creating a local stiffness gradient the cells can mechanically interact with other cells up to 5 cell lengths from their periphery, an effect which allows for long-range pattern formation not seen on linearly elastic materials but common in biological tissues and desirable in engineered constructs.

Many studies of mechanosensing have been performed using NIH 3T3 fibroblasts because this cell line is robust, immortalized, simple to culture and reasonably contractile. Recent studies, however, indicate that immortalized cells may not be representative of how primary cells respond to mechanical stimuli since the expression of cytoskeletal remodeling proteins changes with immortalization [35]. Fewer studies have examined hMSCs but due to their multipotency and large expansion potential [36] they are appealing for tissue engineering and since they display differentiation sensitivity to substrate stiffness [6], understanding their response to the mechanical properties of biological gels is essential for rational biomaterial design. Since both cell types displayed the same trends on fibrin, this sensitivity to nonlinearity may be common in durotactic cells. The capacity of cells to sense and manipulate the nonlinear elasticity of their substrate appears to depend on the degree to which the material strain-stiffens, the inherent contractility of the cell, and the engagement of appropriate integrins.

Two distinct processes contribute to the mechanisms by which a gel formed by filamentous biopolymers strain stiffens. For some biological materials, like fibrin protofibril gels or crosslinked F-actin where the polymer persistence length is on the order of the distance between crosslinks, nonlinear elasticity is thought to result from the intrinsically non-linear force-extension relations of the network strands [11]. For networks with stiffer polymers, like collagen or thick fibrin fibers an additional phenomenon associated with strain stiffening is an increase in local fiber alignment, and a transition from filament bending to filament stretching [37], [38].

One concern is that it is difficult to separate the response to mechanical properties of the gel from responses to structural properties. In the case of initial cell spreading, prior to cell attachment the gel is an isotropic mesh and the onset of alignment in fibrin occurs significantly later than the onset of strain stiffening [39]; therefore isolated cell spreading is likely driven by the gel's mechanical properties. The contribution of the fiber alignment observed at the cell's periphery to the orientation phenomena is unknown, but the tendency for cells to align with grooves on a substrate correlates more with the depth than the width of the grooves, suggesting that enhanced contact area, which simulates a 3D environment, rather than a preference for aligned substrates is the driving force [40]. The filaments and grooves that have been shown to effectively induce contact guidance are 5–20 times larger than the fibrin fibers used in this study [41], [42], [43]. If the topography is too fine it does not supply the increased contact area which promotes contact guidance. The experiments done on aligned fibers less than 100 nm in diameter were performed using biological polymers capable of strain stiffening and did not measure or control for anisotropy in mechanical properties that resulted from the fiber alignment.[44], [45]. It is probable that both the mechanical and structural anisotropy contribute to the observed orientation effect, and both of these features result from the underlying nonlinear response of the fibrin gels to cell-applied strains.

In past experiments on linearly elastic materials, cells applied weaker tractions on softer substrates, and stronger tractions corresponded to larger spread areas [27]. As cells appear to reach their maximum spread area on fibrin gels regardless of initial gel stiffness, it appears that the cell and gel engage in a mechanical “tug-of-war” until one or the other can no longer increase resistance. In the case of polyacrylamide gels the resistance is constant so if the cell can match the gel's resistance, as 3T3 cells can on a gel of 1 kPa, the cell applies that much resistance but no more. This response is indicated by a smaller than maximum spread area and cortical stiffness measured by AFM [46]. In contrast to linear materials, a fibrin gel will increase resistance as the cell applies greater force resulting in a feedback loop that will continue until either the cell or the gel can no longer increase resistance. In all the conditions of fibrin gels tested so far, fibroblasts and hMSCs spread to their maximal areas, suggesting that the gel can stiffen beyond the cell's ability to contract. Figure 5B shows that, as predicted by the tug-of-war hypothesis, the cells applied larger displacements on gels with a lower initial modulus, because cells must apply larger strains to a softer gel to achieve the same final stiffness. This result also indicates that the cell's mechanosensor is stress limited not strain limited which is consistent with a previous study on a collagen substrate [47].

A recent experimental and computation studies reported that cells on moderately compliant synthetic matrices altered the behavior of neighboring cells through traction forces transmitted through the compliant gel [19], [48]. Since the experiments were done using polyacrylamide substrates, where the cell contraction scales with gel stiffness, the reported mechanical communication only occurred if the cells were within 50 microns and large scale pattern formation such as was seen in the system reported here was not observed. This difference underscores why linearly elastic materials might be an incomplete model system for predicting how cells will respond in biological matrices.

The results presented here have implications both for smart design of tissue engineering constructs and for understanding the progression of diseases correlated with changes in tissue stiffness such as cancer [3] and fibrosis [49]. One significant hurdle limiting clinical viability of tissue engineering is finding a way to stably vascularize the construct. A recent study reported that hMSCs stabilized functional engineered vasculature composed of human umbilical cord vein endothelial cells suspended in a gel of fibronectin and collagen [50]. Although mechanics was not explicitly considered, the results from this work suggest that in addition to providing paracrine support, the hMSCs may also facilitate the network formation required for capillary development. The data presented here suggests that when selecting a material to match the stiffness of tissue it is crucial to test the tissue's nonlinear properties to determine the relevant strain at which the moduli should be matched. Lastly, it was recently reported that early stiffening of liver tissue is likely due to an increase in lysyl oxidase mediated crosslinking of the collagen ECM and that this stiffness increase precedes myofibroblast activation and fibrosis in chronic liver disease [51]. Crosslinking not only increases the low strain modulus but, by decreasing the contour length between network junctions, it can also increase the degree of strain stiffening, so the ultimate stiffness felt by the cell is likely higher than reported from low-strain rheology. Further studies need to be done but as differentiation from portal fibroblast to myofibroblast requires both TGF-beta and a stiff substrate [49] an increase in local strain stiffening may be a key step in chronic liver disease.

## Materials and Methods

### Cell Culture

Bone marrow-derived human mesenchymal stem cells (Cambrex) were maintained in DMEM (GIBCO) with 1 g/L D-glucose, 0.3mg/ml L-glutamine and 100 mg/L sodium pyruvate, 100 units/ml penicillin, 100 µg/ml streptomycin and 10% heat inactivated fetal bovine serum (GIBCO) on tissue culture plastic prior to seeding on gels. NIH 3T3 fibroblast's (ATCC) culture media was identical except it contained 4.5 g/L D-glucose and 10% calf serum (GIBCO) instead of fetal bovine serum. All cells were maintained at 37°C and 5% CO_2_. Unless otherwise specified all reagents are analytical grade and purchased from Sigma.

### Fibrin Gel Preparation

Lyophilized salmon fibrinogen [52] and thrombin [53] were provided by Sea Run Holdings. Fibrin gels were prepared by diluting the stock solution of fibrinogen with T7 buffer (50 mM Tris, 150 mM NaCl at a pH of 7.4) to make a working solution of desired concentration. Polymerization was initiated in a 24 well tissue culture plate by adding 5 µl of thrombin (activity = 100 NIH units/ml) to 250 µl of the fibrinogen solution. Gels were allowed to polymerize for 30 minutes before cell culture media was added to the wells. Gel thickness was measured using the z control of the rheometer and found to be approximately 1 mm at the thinnest point. In some cases 0.15 mM glycine-proline-arginine-proline (GPRP) was added to the working solution or the already polymerized gel was incubated in a 0.01 mg/ml solution of type 1 rat tail collagen for four hours at 4°C.

### Polyacrylamide Gel Preparation

Polyacrylamide gels of varying stiffness were prepared as reported previously [54] with the modifications described in [55]. The ligand attached to the surface was one of the following: fibrinogen monomers, fibrin monomers, thin fibrin fibrils, thick fibrin fibrils, a network of thin fibers or a network of thick fibers. Fibrinogen monomers were ligated to the gel at a concentration of 0.1 mg/ml for 1.5 hours. To achieve the coating of fibrin monomers, the gel was first coated with fibrinogen monomers, washed with T7 buffer and then immersed in a solution of 1 U/ml thrombin for 30 minutes. For the two network coated substrates, the polyacrylamide was first ligated to fibrinogen monomers which act as nucleation sites and then coated with either 0.1 mg/ml fibrinogen and 0.5 U/ml thrombin for 30 minutes or with 0.5 mg/ml fibrinogen and 0.5 U/ml thrombin for 30 minutes. The networks were 5–10 fibers high and less than 2 microns thick. The thin fibers were produced by preparing a solution of 0.1 mg/ml fibrinogen and 0.1 U/ml thrombin 1 hour in advance and then allowing it to react with the gel for 1.5 hours. The thick fibers were prepared by allowing a solution of 0.5 mg/ml fibrinogen to react with 0.2 U/ml thrombin for 2 minutes and then adding excess of the thrombin inhibitor p-nitrophenyl-p'-guanidinobenzoate. This solution of fibers was then reacted with the surface of the polyacrylamide for 1.5 hours.

### Fibrin Microscopy

Untreated fibrinogen was spiked with biotinylated fibrinogen (1∶50), the gels were polymerized as usual and then the surface was coated with streptavidin-coated red fluorescent beads (1000 beads/µm^2^, Invitrogen). Biotin-NHS (Sigma) was conjugated to salmon fibrinogen using the manufacturer's protocol. Cells were seeded on fluorescent bead-coated fibrin gels (100 cells/well) and allowed to adhere and spread for 18 hours. In some cases the broad spectrum matrix metalloproteases inhibitor GM 6001 (Calbiochem) at a concentration of 20 µM was added to the cell culture media prior to seeding the cells. For imaging, the cells were kept in serum-free media buffered with 50 mM HEPES and the microscope stage was heated to 37°C. Cells were imaged in phase contrast mode and the beads were imaged in fluorescence before the treatment (1% DMSO, 5 µM blebbistatin, 20 µM Cytochalasin D or 10 µM Nocodazole) was added. After 30 minutes the beads were imaged again. Image J (NIH) and Adobe Photoshop were used to generate a map of bead displacements for each pair of images. A blinded assistant determined the total bead displacement within a particular area, which was then averaged over the total number of beads in that area. Due to the low magnification needed to capture the scale of the gel deformation the pixel resolution resulted in a error of±0.25 µm. To minimize the effect of cells other than the one of interest, only 50–100 cells were seeded per well of the 24 well dish. In addition the imaged cells were selected because they were at least 1 mm from the nearest cell.

### Rheology Measurements

To measure the dynamic shear storage modulus (G') of fibrin gels, 300 µl of fibrin with or without cells were prepared in a 24 well plate. These gels were prepared identically to the microscopy gels except that the fibrinogen was diluted to 2 mg/ml in a cell suspension or just cell type specific media. The gels were cultured overnight at 37°C to allow sufficient time for gel contraction. The shear modulus was calculated from the in-phase shear stress on a strain-controlled RFS III fluids spectrometer rheometer (Rheometrics, Piscataway, NJ) with an 8 mm parallel plate geometry on a stage heated to 37°C using a 2% oscillatory shear strain at a frequency of 5 radians per second. These parameters were chosen to probe the elastic response of the gels in the low strain region where G' is independent of strain. To measure the strain stiffening response, gels were polymerized between 25 mm parallel plates for 10 minutes at which time silicone oil was added to the perimeter to prevent drying and the gels were allowed to polymerize for another 50 minutes on a Bowlin Gemini rheometer. The strain was then ramped from 0 to 100% strain at 5 radians per second and 10 points per decade. The resisting stress and estimated elastic modulus were measured at each point.

### Atomic Force Microscopy

Force indentation curves of fibrin gels were performed as previously described [46] on a Veeco Bioscope I using a silicon nitride probe with a cantilever spring constant of 0.01 N/m and a 5 µm polystyrene particle attached. Relative stiffnesses of different points on the gel are estimated by fitting the first 500 nm of the indentation curves to the Hertz model [56].

### Image Analysis

All light imaging was done on a Leica DMIRE microscope using a Hamamatsu ORCA-ER camera and a 10× air lens with a numerical aperture of 0.30 or a 40× air lens with a numerical aperture of 0.60. With the exception of the displacement images, analysis was done on images of cells fixed and stained with phalloidin and DAPI. Image J software was used to convert images to 8 bit, and then threshold and analyze the cells' projected area, alignment and distance to the nearest cell. Cell projected area was determined using Image J's analyze particles command. Cell alignment was determined by using Image J to fit an ellipse to a cell and then using the angle tool to measure the angle between the cell's long access and the membrane of the nearest cell with the vertex at the cell's center of mass. Axial ratio was determined by using ImageJ to fit an ellipse to a cell and then taking the ratio of the long axis to the short axis. Distance from membrane to membrane of the nearest cell was determined using the ruler tool. DAPI staining allowed verification that only individual non-dividing cells were counted.

### Statistics

All statistics was preformed using Kaleidagraph software and unless otherwise mentioned significance was determined using a one way ANOVA with and then applying a Tukey HSD post test with an α threshold of 0.05

## Supporting Information

Text S1 Methods for supplemental figures.
(0.02 MB DOC)

Figure S1 Ligand coverage varies by polyacrylamide coating. Representative scanning electron micrographs of polyacrylamide coated surfaces: (A) fibrinogen monomers, (B) fibrin monomers, (C) non contiguous covering thin fibrin fibers, (D) non contiguous covering of thick fibrin fibers, (E) a network of thin fibers, or (F) a network of thick fibers.
(2.73 MB TIF)

Figure S2 Fiber in fibrin gel are randomly oriented prior to cell attachment and become aligned and bundled perpendicular to a spread cell's membrane. Tapping mode AFM images of a cell free 2 mg/ml fibrin gel (A) and a section of gel adjacent to a spread hMSC (B). The edge of the cell's membrane is just off the left side of frame B. The gradient scale bar reports the sample height
(1.07 MB TIF)

Figure S3 Axial ratio is dependent on fibrinogen concentration but independent of distance to the nearest cell. The axial ratio of neighboring hMSCs were analyzed as a function of distance to the nearest cell on four substrates: 1, 2, and 8 mg/ml fibrin as well as 16 kPa fibrinogen coated polyacrylamide. Reported as mean±SE, n = 3, at least 10 cells per group.
(0.72 MB TIF)

## References

[1] Yeung T, Georges PC, Flanagan LA, Marg B, Ortiz M (2005). Effects of substrate stiffness on cell morphology, cytoskeletal structure, and adhesion.. Cell Motil Cytoskeleton.

[2] Pelham RJ, Wang YL (1998). Cell locomotion and focal adhesions are regulated by the mechanical properties of the substrate.. Biol Bull.

[3] Paszek MJ, Zahir N, Johnson KR, Lakins JN, Rozenberg GI (2005). Tensional homeostasis and the malignant phenotype.. Cancer Cell.

[4] Georges PC, Miller WJ, Meaney DF, Sawyer ES, Janmey PA (2006). Matrices with compliance comparable to that of brain tissue select neuronal over glial growth in mixed cortical cultures.. Biophys J.

[5] Fereol S, Fodil R, Labat B, Galiacy S, Laurent VM (2006). Sensitivity of alveolar macrophages to substrate mechanical and adhesive properties.. Cell Motil Cytoskeleton.

[6] Engler AJ, Sen S, Sweeney HL, Discher DE (2006). Matrix elasticity directs stem cell lineage specification.. Cell.

[7] Wang YL, Pelham RJ (1998). Preparation of a flexible, porous polyacrylamide substrate for mechanical studies of cultured cells.. Methods Enzymol.

[8] Reinhart-King CA, Dembo M, Hammer DA (2003). Endothelial Cell Traction Forces on RGD-Derivatized Polyacrylamide Substrata&#x2020.. Langmuir.

[9] Harris AK, Wild P, Stopak D (1980). Silicone rubber substrata: a new wrinkle in the study of cell locomotion.. Science.

[10] Tan JL, Tien J, Pirone DM, Gray DS, Bhadriraju K (2003). Cells lying on a bed of microneedles: an approach to isolate mechanical force.. Proc Natl Acad Sci U S A.

[11] Storm C, Pastore JJ, MacKintosh FC, Lubensky TC, Janmey PA (2005). Nonlinear elasticity in biological gels.. Nature.

[12] Janmey PA, McCormick ME, Rammensee S, Leight JL, Georges PC (2007). Negative normal stress in semiflexible biopolymer gels.. Nat Mater.

[13] Pelham RJ, Wang Y (1999). High resolution detection of mechanical forces exerted by locomoting fibroblasts on the substrate.. Mol Biol Cell.

[14] Saez A, Buguin A, Silberzan P, Ladoux B (2005). Is the mechanical activity of epithelial cells controlled by deformations or forces?. Biophys J.

[15] Brown AEX, Litvinov RI, Discher DE, Weisel JW (2007). Forced unfolding of the coiled-coils of fibrinogen by single molecule AFM.. Biophysical Journal.

[16] Collet JP, Shuman H, Ledger RE, Lee S, Weisel JW (2005). The elasticity of an individual fibrin fiber in a clot.. Proc Natl Acad Sci U S A.

[17] Lim BBC, Lee EH, Sotomayor M, Schulten K (2008). Molecular basis of fibrin clot elasticity.. Structure.

[18] Kolodney MS, Elson EL (1995). Contraction due to microtubule disruption is associated with increased phosphorylation of myosin regulatory light chain.. Proc Natl Acad Sci U S A.

[19] Reinhart-King CA, Dembo M, Hammer DA (2008). Cell-cell mechanical communication through compliant substrates.. Biophys J.

[20] Dembo M, Wang YL (1999). Stresses at the cell-to-substrate interface during locomotion of fibroblasts.. Biophys J.

[21] Schindlauer G, Bale MD, Ferry JD (1986). Interaction of fibrinogen-binding tetrapeptides with fibrin oligomers and fine fibrin clots.. Biopolymers.

[22] Hiraoka N, Allen E, Apel IJ, Gyetko MR, Weiss SJ (1998). Matrix metalloproteinases regulate neovascularization by acting as pericellular fibrinolysins.. Cell.

[23] Hotary KB, Yana I, Sabeh F, Li XY, Holmbeck K (2002). Matrix metalloproteinases (MMPs) regulate fibrin-invasive activity via MT1-MMP-dependent and -independent processes.. J Exp Med.

[24] Leitinger B, Hohenester E (2007). Mammalian collagen receptors.. Matrix Biol.

[25] Hong H, Stegemann JP (2008). 2D and 3D collagen and fibrin biopolymers promote specific ECM and integrin gene expression by vascular smooth muscle cells.. J Biomater Sci Polym Ed.

[26] Weisel JW (2005). Fibrinogen and fibrin.. Adv Protein Chem.

[27] Lo CM, Wang HB, Dembo M, Wang YL (2000). Cell movement is guided by the rigidity of the substrate.. Biophys J.

[28] Balaban NQ, Schwarz US, Riveline D, Goichberg P, Tzur G (2001). Force and focal adhesion assembly: a close relationship studied using elastic micropatterned substrates.. Nat Cell Biol.

[29] Vailhe B, Ronot X, Tracqui P, Usson Y, Tranqui L (1997). In vitro angiogenesis is modulated by the mechanical properties of fibrin gels and is related to alpha(v)beta3 integrin localization.. In Vitro Cell Dev Biol Anim.

[30] Deroanne CF, Lapiere CM, Nusgens BV (2001). In vitro tubulogenesis of endothelial cells by relaxation of the coupling extracellular matrix-cytoskeleton.. Cardiovasc Res.

[31] Sieminski AL, Hebbel RP, Gooch KJ (2004). The relative magnitudes of endothelial force generation and matrix stiffness modulate capillary morphogenesis in vitro.. Exp Cell Res.

[32] Bischofs IB, Schwarz US (2003). Cell organization in soft media due to active mechanosensing.. Proc Natl Acad Sci U S A.

[33] Bridgman PC, Dave S, Asnes CF, Tullio AN, Adelstein RS (2001). Myosin IIB is required for growth cone motility.. J Neurosci.

[34] Chan CE, Odde DJ (2008). Traction dynamics of filopodia on compliant substrates.. Science.

[35] Alge CS, Hauck SM, Priglinger SG, Kampik A, Ueffing M (2006). Differential protein profiling of primary versus immortalized human RPE cells identifies expression patterns associated with cytoskeletal remodeling and cell survival.. J Proteome Res.

[36] Kassem M (2004). Mesenchymal stem cells: biological characteristics and potential clinical applications.. Cloning Stem Cells.

[37] Huisman EM, van Dillen T, Onck PR, Van der Giessen E (2007). Three-dimensional cross-linked F-actin networks: relation between network architecture and mechanical behavior.. Phys Rev Lett.

[38] Onck PR, Koeman T, van Dillen T, van der Giessen E (2005). Alternative explanation of stiffening in cross-linked semiflexible networks.. Phys Rev Lett.

[39] Kang H, Wen Q, Janmey P, Tang J, Conti E (2009). Non-linear elasticity of stiff filament networks: Strain stiffening, negative normal stress, and filament alignment in fibrin gels.. J Phys Chem B: in press.

[40] Clark P, Connolly P, Curtis AS, Dow JA, Wilkinson CD (1991). Cell guidance by ultrafine topography in vitro.. J Cell Sci.

[41] Walboomers XF, Monaghan W, Curtis AS, Jansen JA (1999). Attachment of fibroblasts on smooth and microgrooved polystyrene.. J Biomed Mater Res.

[42] Xu CY, Inai R, Kotaki M, Ramakrishna S (2004). Aligned biodegradable nanofibrous structure: a potential scaffold for blood vessel engineering.. Biomaterials.

[43] Yim EK, Reano RM, Pang SW, Yee AF, Chen CS (2005). Nanopattern-induced changes in morphology and motility of smooth muscle cells.. Biomaterials.

[44] Dubey N, Letourneau PC, Tranquillo RT (2001). Neuronal contact guidance in magnetically aligned fibrin gels: effect of variation in gel mechano-structural properties.. Biomaterials.

[45] Lee P, Lin R, Moon J, Lee LP (2006). Microfluidic alignment of collagen fibers for in vitro cell culture.. Biomed Microdevices.

[46] Solon J, Levental I, Sengupta K, Georges PC, Janmey PA (2007). Fibroblast adaptation and stiffness matching to soft elastic substrates.. Biophys J.

[47] Freyman TM, Yannas IV, Yokoo R, Gibson LJ (2002). Fibroblast contractile force is independent of the stiffness which resists the contraction.. Exp Cell Res.

[48] Sen S, Engler AJ, Discher DE (2009). Matrix strains induced by cells: Computing how far cells can feel.. Cellular and Molecular Biology.

[49] Li Z, Dranoff JA, Chan EP, Uemura M, Sevigny J (2007). Transforming growth factor-beta and substrate stiffness regulate portal fibroblast activation in culture.. Hepatology.

[50] Au P, Tam J, Fukumura D, Jain RK (2008). Bone marrow-derived mesenchymal stem cells facilitate engineering of long-lasting functional vasculature.. Blood.

[51] Georges PC, Hui JJ, Gombos Z, McCormick ME, Wang AY (2007). Increased stiffness of the rat liver precedes matrix deposition: implications for fibrosis.. Am J Physiol Gastrointest Liver Physiol.

[52] Wang LZ, Gorlin J, Michaud SE, Janmey PA, Goddeau RP (2000). Purification of salmon clotting factors and their use as tissue sealants.. Thromb Res.

[53] Michaud SE, Wang LZ, Korde N, Bucki R, Randhawa PK (2002). Purification of salmon thrombin and its potential as an alternative to mammalian thrombins in fibrin sealants.. Thromb Res.

[54] Pelham RJ, Wang Y (1997). Cell locomotion and focal adhesions are regulated by substrate flexibility.. Proc Natl Acad Sci U S A.

[55] Winer JP, Janmey PA, McCormick ME, Funaki M (2009). Bone Marrow-Derived Human Mesenchymal Stem Cells Become Quiescent on Soft Substrates but Remain Responsive to Chemical or Mechanical Stimuli.. Tissue Eng Part A.

[56] Hertz HJ (1882). Über die Berührung fester elastischer Körper.. J Reine Angew.

[57] Langer BG, Weisel JW, Dinauer PA, Nagaswami C, Bell WR (1988). Deglycosylation of fibrinogen accelerates polymerization and increases lateral aggregation of fibrin fibers.. J Biol Chem.

