# Supplementary material for: Non-Linear Elasticity of Extracellular Matrices Enables Contractile Cells to Communicate Local Position and Orientation
Source: PLoS One. 2009 Jul 24;4(7):e6382. doi: 10.1371/journal.pone.0006382 (PMC2711623; doi:10.1371/journal.pone.0006382)
Supplement: Text S1 — Methods for supplemental figures. (0.02 MB DOC) [file pone.0006382.s001.doc]

Supplementary Materials and Methods:

Scanning Electron Microscopy

For scanning electron microscopy samples were fixed with 2% glutaraldehyde in sodium cacodylate buffer, dehydrated with ethanol, critical point dried and coated with gold/palladium as described previously[1].  The images were taken on a Philips XL20 scanning electron microscope (Philips Electron Optics, Eindhoven, The Netherlands) in the electron microscopy facility at the Department of Cell Biology University of Pennsylvania, PA, USA.

Imaging with Atomic Force Microscopy

Samples were fixed and dehydrated using the same methods for SEM sample preparation. The samples were the immersed in 50% ethanol, 50% hexamethyldisilazane (HMDS) for 10 minutes and then immersed in 100% HMDS for 10 minutes. The excess solution was removed and the samples were placed in a vacuum chamber for 1 hr to facilitate evaporation of the HMDS. HMDS dried samples were then imaged in tapping mode on a Veeco multimode AFM with pyramid tip silicon probes with a cantilever spring constant of 3 N/m.

1. Langer BG, Weisel JW, Dinauer PA, Nagaswami C, Bell WR (1988) Deglycosylation of fibrinogen accelerates polymerization and increases lateral aggregation of fibrin fibers. J Biol Chem 263: 15056-15063.
