# Supplementary figures and images for: Non-Linear Elasticity of Extracellular Matrices Enables Contractile Cells to Communicate Local Position and Orientation
Source: PLoS One. 2009 Jul 24;4(7):e6382. doi: 10.1371/journal.pone.0006382 (PMC2711623; doi:10.1371/journal.pone.0006382)

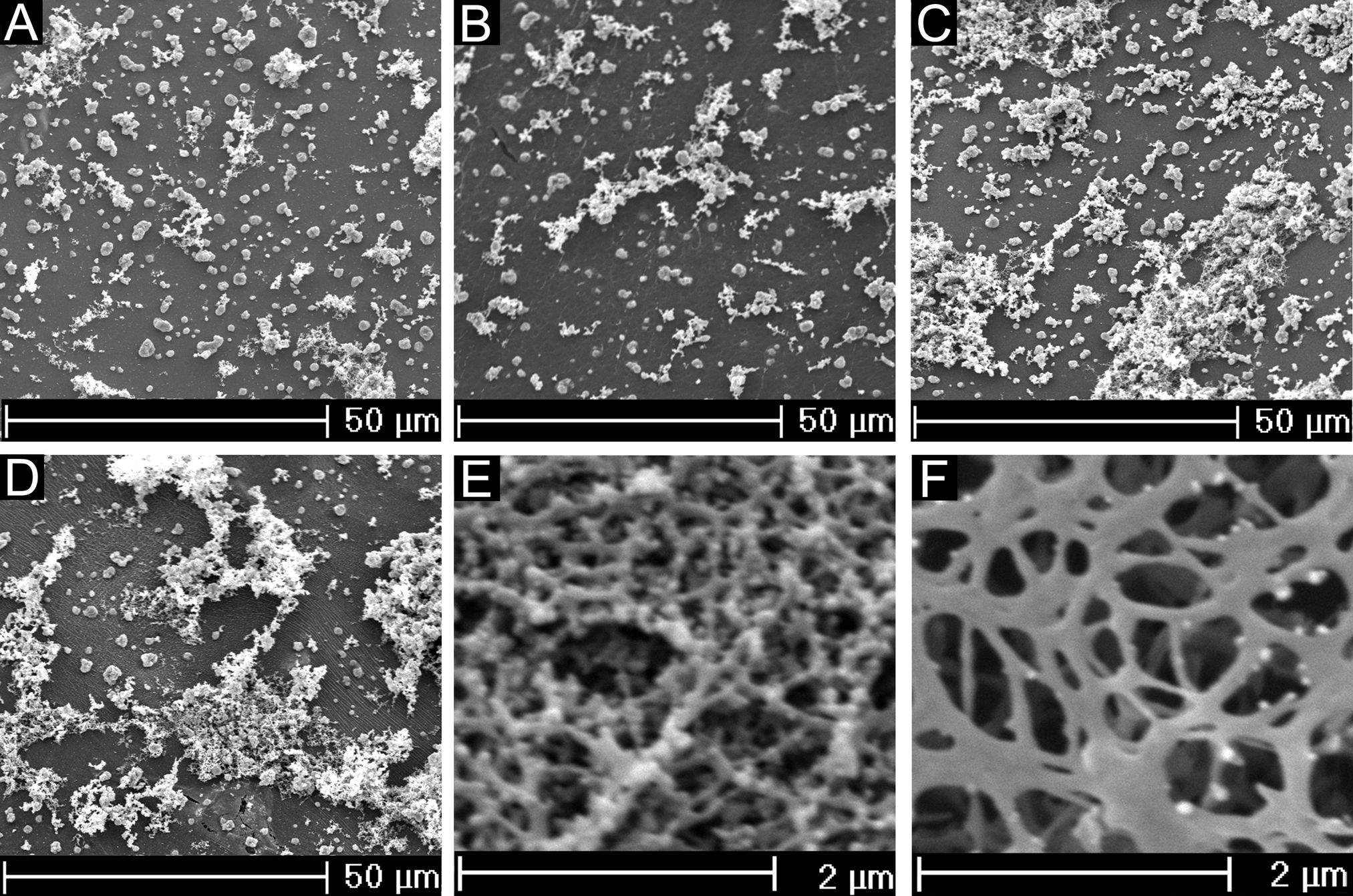

Supplement: Figure S1 — Ligand coverage varies by polyacrylamide coating. Representative scanning electron micrographs of polyacrylamide coated surfaces: (A) fibrinogen monomers, (B) fibrin monomers, (C) non contiguous covering thin fibrin fibers, (D) non contiguous covering of thick fibrin fibers, (E) a network of thin fibers, or (F) a network of thick fibers. (2.73 MB TIF) [file pone.0006382.s002.tif]

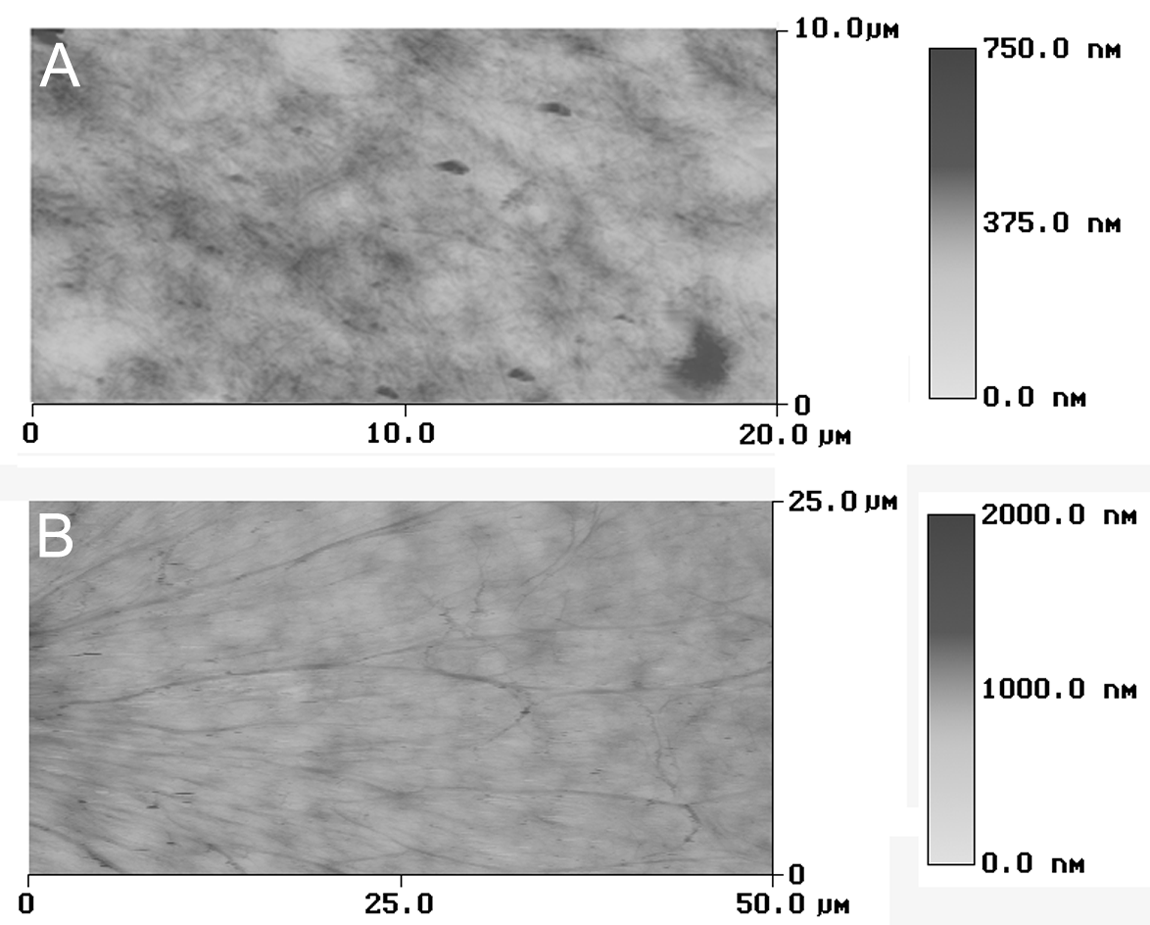

Supplement: Figure S2 — Fiber in fibrin gel are randomly oriented prior to cell attachment and become aligned and bundled perpendicular to a spread cell's membrane. Tapping mode AFM images of a cell free 2 mg/ml fibrin gel (A) and a section of gel adjacent to a spread hMSC (B). The edge of the cell's membrane is just off the left side of frame B. The gradient scale bar reports the sample height (1.07 MB TIF) [file pone.0006382.s003.tif]

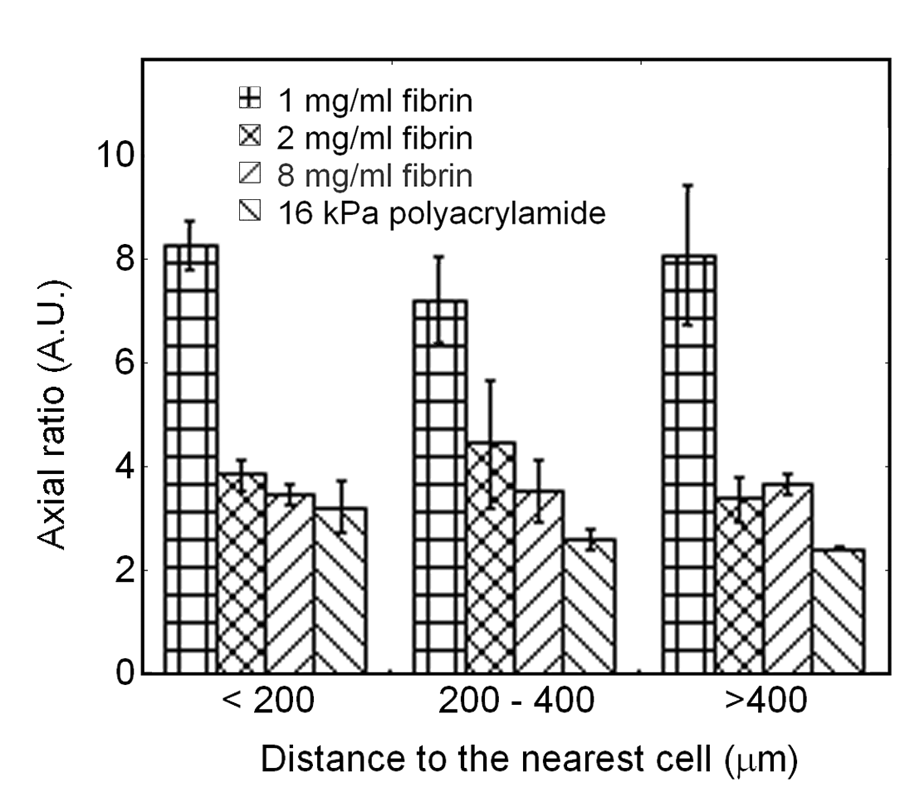

Supplement: Figure S3 — Axial ratio is dependent on fibrinogen concentration but independent of distance to the nearest cell. The axial ratio of neighboring hMSCs were analyzed as a function of distance to the nearest cell on four substrates: 1, 2, and 8 mg/ml fibrin as well as 16 kPa fibrinogen coated polyacrylamide. Reported as mean±SE, n = 3, at least 10 cells per group. (0.72 MB TIF) [file pone.0006382.s004.tif]
